# A model explaining refugee experiences of the Australian healthcare system: a systematic review of refugee perceptions

**DOI:** 10.1186/s12914-019-0206-6

**Published:** 2019-07-18

**Authors:** Michael Au, Athire Debbie Anandakumar, Robyn Preston, Robin A. Ray, Meg Davis

**Affiliations:** 1 College of Medicine and Dentistry, James Cook University, Townsville, Queensland Australia; 2 School of Health, Medical and Applied Sciences, CQUniversity, Townsville, Queensland Australia; 3 Townsville Multicultural Support Group Incorporated, Townsville, Queensland Australia

**Keywords:** Refugees, Perception, Access, Engagement, Qualitative, Health services, Trust, Power, Health information, Autonomy, Cultural competency, Public health

## Abstract

**Background:**

Refugees have significant unmet health needs. Delivering services to refugees continues to be problematic in the Australian healthcare system. A systematic review and thematic synthesis of the literature exploring refugee perceptions of the Australian healthcare system was performed.

**Methods:**

Titles and abstracts of 1610 articles published between 2006 and 2019 were screened, and 147 articles were read in full text. Depending on the type of study, articles were appraised using the Modified Critical Appraisal Tool (developed by authors), the Mixed Methods Appraisal Tool, or the JBI Appraisal Checklist for Systematic Reviews. Using QSR NVivo 11, articles were coded into descriptive themes and synthesised into analytical themes. An explanatory model was used to synthesise these findings. Confidence in the review findings were assessed with GRADE-CERQual approach.

**Results:**

The final synthesis included 35 articles consisting of one systematic review, 7 mixed methods studies, and 27 qualitative studies. Only one study was from a regional or rural area. A model incorporating aspects of engagement, access, trust, and privacy can be used to explain the experiences of refugees in using the Australian healthcare system. Refugees struggled to engage with health services due to their unfamiliarity with the health system. Information sharing is needed but this is not always delivered effectively, resulting in disempowerment and loss of autonomy. In response, refugees resorted to familiar means, such as family members and their pre-existing cultural knowledge. At times, this perpetuated their unfamiliarity with the broader health system. Access barriers were also encountered. Trust and privacy are pervasive issues that influenced access and engagement.

**Conclusions:**

Refugees face significant barriers in accessing and engaging with healthcare services and often resorted to familiar means to overcome what is unfamiliar. This has implications across all areas of service provision. Health administrators and educators need to consider improving the cultural competency of staff and students. Policymakers need to consider engaging communities and upscale the availability and accessibility of professional language and cultural supports. Research is needed on how these measures can be effectively delivered. There is limited research in remote areas and further evidence is needed in these settings.

**Electronic supplementary material:**

The online version of this article (10.1186/s12914-019-0206-6) contains supplementary material, which is available to authorized users.

## Background

Refugees in Australia are consistently recognised as an underserved population with higher rates of mental health and infectious diseases [1–3]. This is compounded by pre-arrival and post-arrival factors including poor care in their country of origin, trauma, prolonged detention, and barriers to appropriate care on arrival [4].

Seeking refugee status in Australia is tightly controlled. In the year 2016–2017, 21,968 visas were granted under the Refugee and Humanitarian Assistance Programme including 8208 places for displaced Syrian and Iraqi refugees. Those who arrive in Australia without a visa are subject to mandatory detention [5]. The evidence indicates a clear detrimental effect of indefinite detention, especially on mental health, and the morbidity is transferred into settlement [6–9]. Given these health issues, most States or Territories have their own policies with different targets and objectives towards improving refugee health [10–12]. However, to date, there is still no coordinated national policy.

Once in the community, refugees have access to Medicare Benefit Schedule item numbers that allows a general practitioner to complete a refugee health assessment within the first 12 months [13]. Refugees are also linked with resettlement agencies that provide some assistance in navigating the health system for six to 18 months upon arrival [14]. These services shape refugees’ initial experiences with the healthcare system.

Current literature exploring some of the challenges and facilitators faced by health professionals in delivering primary healthcare for refugees and asylum seekers in high-income countries can be conceptualised into three broad themes: the healthcare encounter, working within the healthcare system, and asylum and resettlement [15]. However, research is needed to understand the experiences of refugees as an integral part of a framework to provide effective solutions to address these barriers. Furthermore, studies that collectively group high-income countries together do not adequately address the unique geographical profile, health system characteristics and social profile of Australia. These all play a role in health, particularly, rural and remoteness. This is of significance as there have been efforts made by the Australian Government to resettle refugees in regional areas [16].

From the best available knowledge, no other systematic review has examined refugee perceptions of using Australian healthcare services. A scoping review of refugee perceptions in their host country only included two Australian studies with other international data [17]. A similar review examined refugee experiences of general practice in their countries of resettlement. However, the article was restricted to general practice [18]. Other primary research have considered refugee experiences of healthcare services but this is often limited to one particular service or setting [19, 20]. Examining the experiences of Australian refugees may be beneficial in an international context for nations that resettle refugees in regional and remote areas as well as those countries that offer universal primary healthcare. Considering the current state of the literature, the aim of this study was to explore the perceptions of refugees in using Australian healthcare services. Articles published from 2006 to 2019 were selected to capture the current refugee demographics that occurred post-Iraq and Afghanistan conflicts. It also reflects Australia’s most recent refugee intake after the end of the Pacific Solution policy in 2007.

## Methods

### Protocol and registration

This review adopted a thematic synthesis approach and adhered to ENTREQ and PRISMA guidelines [21–23]. The review was registered with PROSPERO (registration number: CRD42018088364).

### Eligibility criteria

*Time frame*: Studies published between the years 2006 to 2019 were included to capture Australia’s most recent refugee demographics after the Iraq and Afghanistan conflicts.

*Population:* Refugees in Australia. Asylum seekers, immigrants, migrants, and displaced persons were excluded. Studies that had refugees as part of a heterogenous population were included if it clearly stated that some participants were refugees.

*Language*: English language only.

*Intervention:* Articles had to relate to refugee interaction with a specific health service or health intervention. Health seeking behaviours or service utilisation were insufficient to judge perceptions of using a health service and therefore excluded.

*Outcomes*: Data related to the perception of refugees using health services. Studies that included the perceptions of refugees and health service providers were considered but only data relating to refugees were reviewed.

*Types of articles:* Qualitative, quantitative, mixed methods, systematic reviews, and grey literature were included. Letters, commentaries and case studies were excluded. For systematic reviews, only the findings from studies relating to refugees were considered.

### Information sources

Studies were identified through electronic databases including Scopus, CINHAL, PubMed, MEDLINE, Cochrane, and Informit. Hand searching was also used to select studies. Geographical limits to Australia were applied to all the databases where available. The last search was ran on the 2nd of April 2019.

### Search

The search strategy is presented in Additional file 2. Maintaining close adherence to the search terms for multiple databases was ensured. As there is some variability in the definition of refugees, asylum seekers, migrants, and immigrants, all these terms were included in the search strategy to avoid any missed articles.

### Study selection

Duplicates were first removed, and articles published outside 2006 to 2019 were excluded. Two researchers (MA and AA) independently screened the articles, first by title, then abstract. Full text articles were screened. One author had to be contacted to retrieve full text. At each stage of the process, eligibility was negotiated by consensus. When consensus was not met, a third researcher (RP) was involved to decide its selection.

### Data collection process, data items, and analysis

Two researchers (MA and AA) were involved in the data extraction process using QSR NVivo 11 software. Coding was regularly reviewed by authors to improve inter-coder reliability. Disagreements were resolved through consensus. For studies that had a heterogenous population involving participants other than just refugees, only the data that was related to refugees was coded. Where it was not clear to assessors if certain data related to refugees, data was included for analysis, but this affected its appraisal performance and the confidence in the review findings.

Both first order and second order constructs were included in the extraction process to capture the author’s interpretation [24, 25]. Line-by-line coding relating to refugee perceptions developed the descriptive themes [21]. All authors interpreted the descriptive themes to develop the analytical themes that went beyond the primary studies. An interpretative approach was taken on the collective data whilst ensuring the author’s original interpretation was captured in individual studies [26].

Two researchers (MA and AA) also independently extracted the study characteristics including the aim, methodologies used, study setting, number of refugee participants, gender of participants, country of origin, and services explored.

### Appraisal of articles

Given the lack of consensus over a standardised qualitative appraisal tool, a Modified Critical Appraisal Tool (MCAT) was developed using components of Joanna Briggs Institute (JBI) Critical Appraisal Checklist for Qualitative Research, Critical Appraisal Skills Programme Qualitative Appraisal Checklists, and McMaster University Critical Review Form, in order to capture the breadth and depth of assessment made by different tools (Additional file 1) [27]. The tool assessed for theoretical congruity, fundamentals, credibility, dependability, reporting, and utility. Reporting was assessed using Standards for Reporting Qualitative Research. The MCAT was not assigned a scoring system and a judgment was applied to each component.

The Mixed Methods Appraisal Tool (MMAT) was used for quantitative and mixed methods studies. The qualitative component of mixed methods studies were additionally appraised with the MCAT. Systematic reviews were appraised with the JBI Appraisal Checklist for Systematic Reviews. Consistency across tools was maintained as judgments were applied on components rather than assigning scores or grades. Two researchers (MA and AA) independently appraised the articles and consensus was met on all the articles.

### Assessment of confidence in the review findings

The Confidence in the Evidence from Reviews of Qualitative research (GRADE-CERQual) method was used to assess the confidence of review findings despite the review included quantitative, mixed methods and other systematic reviews. Fidelity to the GRADE-CERQual approach was maintained by adhering to definitions and using the four categories of grading. However, the authors acknowledge potential for this to distort the findings, which GRADE-CERQual assessment cannot assess. However, given the small number of these articles, the degree of alteration is minimal. Two researchers (MA and AA) performed this assessment under the supervision of other authors. Disagreements were resolved through consensus.

## Results

### Study selection

**Fig. 1:**
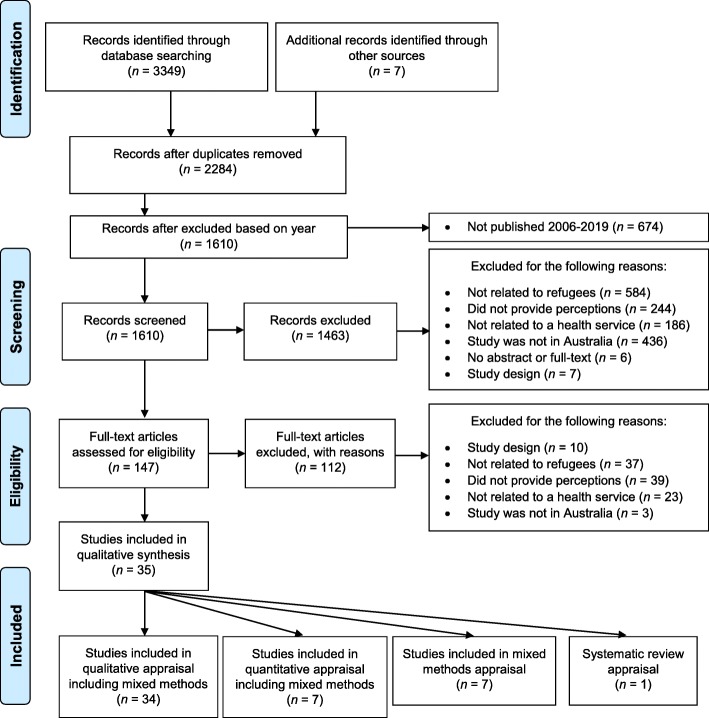
Adapted PRISMA 2009 Flow Diagram

A total of 35 studies were included comprising of one systematic review, seven mixed methods studies, and 27 qualitative studies (see Fig. 1).

The initial search strategy generated 3349 articles with an additional seven articles identified from the reference lists of included articles. A total of 147 articles were read in full text excluding a further 112 articles.

### Study characteristics

Study characteristics and results of individual studies are detailed in Table 1.

**Table 1:** Table of Results. Number of refugees marked with^a^ indicate that it is a mixed population

Study	Study Design	Aim	Study setting	Service Explored	Study Methods	Number of Refugees	Country of Origin or Background	Main Findings
Bellamy et al. 2017[28]	Qualitative	African refugees’ experiences of barriers in accessing pharmacy services in Brisbane.	Brisbane, Queensland	Pharmacy services	Focus groups	16	Somalia (5) Congo (5) South Sudan (2) Uganda (1) Burundi (1) Liberia (1) Eritrea (1)	When describing their experiences in accessing pharmacy services, refugees noted four overarching themes: health system differences, navigating the Australian health system, communication barriers and health care-seeking behaviour.
Carolan et al. 2010[29]	Qualitative	Experiences of African-born pregnant women receiving antenatal care in Melbourne.	Melbourne’s western suburbs	Maternity services	In-depth interviews	18	Ethiopia (1) Sudan (12) Eritrea (2) Somalia (2) Kenya (1)	Five themes that African refugee women giving birth encounter: pregnancy is not special; resettlement is a priority; childbearing is a normal process; coming to value continuous pregnancy care; and cultural sensitivity is important.
Cheng et al. 2015[30]	Qualitative	Factors influencing Afghan refugees’ access at a single general practice in south-east Melbourne in 2013.	City of Greater Dandenong and City of Casey, Victoria	Primary care	Semi-structured interviews and field observation	6	Afghanistan (6)	Barriers to healthcare for newly arrived refugees include language and cultural responsiveness, appointments, difficulties with transport to the practice, long wait times and the cost of care.
Clark et al. 2014[31]	Qualitative	Barriers to accessing primary health care services and exploration of medicine-related issues as experienced by refugee women in South Australia.	South Australia	Using health services and medicines while living in Australia	Focus groups	38	Sudan, Burundi, Congo (15) Burma (10) Afghanistan (5) Bhutan (8)	Patients found that language barriers were the main barrier to accessing primary health care. Interpreters were used inconsistently, and patients noted poor literacy.
Correa-Velez et al. 2012[32]	Mixed methods	Developing a model of refugee maternity healthcare for from consultations with stakeholders, chart audit and surveys with health service providers and refugees.	Mater Mothers’ Hospital, Queensland	Maternity services	Chart audit and surveys (separate surveys with hospital staff)	23	Sudan (14) Burundi (5) Ethiopia (2) Congo (1) Somalia (1)	Participants stated the need for interpreters, education programs for pregnant women, and continuity of care.
Henderson et al. 2011[33]	Qualitative	Access and use of health services by four CALD communities in Logan, Queensland.	Logan, Queensland	All services	Focus groups	42^a^	Sudan, Afghanistan, Pacific Islands and Myanmar	Unfamiliarity with health services, difficulties accessing care were experienced by CALD communities. CALD communities valued traditional medical practices and wanted health practitioners to respect this. Language barriers and interpreter issues prominent.
Kay et al. 2016[34]	Qualitative	Barriers and facilitators of quality use of medicines for from primary healthcare providers and refugee health leaders in Brisbane.	Brisbane, Queensland	Pharmacy services	Semi-structured interviews	3	Sudan (1) Myanmar (1) Liberia (1)	Five barriers were identified between HCWs and refugee health leaders: communication and language constraints, cultural issues, limited health literacy, financial cost, and health system concerns.
Liamputtong et al. 2006[35]	Qualitative	Experience of caesarean birth among Cambodian, Lao and Vietnamese women.	Melbourne, Victoria	Maternity services	In-depth interviews	91^a^	Laos (30) Vietnam (30) Cambodia (31)	Women’s experiences in caesarean birth have three main themes: trust in medical knowledge, expectations and communication with an understanding of their caregivers’ preferences.
McBride et al. 2016[36]	Mixed methods	Evaluation of the Refugee Health Nurse Liaison role at Dandenong Hospital.	Dandenong Hospital, Victoria	Refugee Health Nurse Liaison	Semi-structured surveys (60) and chart audits (journals were only used with service providers)	60	Afghanistan (30) Sri Lanka (12) Iran (6) Burma (2) Iraq (2) Sudan (1) Pakistan (3) Other (2)	Patients noted that Refugee Health Nurse Liaisons were helpful in interpreting and providing helpful information.
McBride et al. 2017[37]	Mixed Methods	Experiences of refugees in using Monash Health Refugee Health and Wellbeing services.	South-East Region of Melbourne	All services	Semi-structured interviews (18) and surveys (159)	177	Afghanistan (77) Sri Lanka (43) Iran (11) Myanmar (16) Iraq (3) Pakistan (14) Bangladesh (7) Other (6)	Refugees were highly satisfied with the service emphasising the importance of a trusting relationship with staff, access to bicultural support workers, onsite interpreting and integrated care.
McCann et al. 2016[38]	Qualitative	Help-seeking barriers and facilitators of mental health and substance use services in recently arrived young Sub-Saharan African migrants in Melbourne.	Melbourne, Victoria	Mental health and substance use services	In-depth interviews (28) and focus groups (41)	69^a^	Sub-Saharan Africa	Participants noted four help-seeking barriers that prevented them from seeking help for mental health and alcohol and drug use: stigma of mental illness, lack of mental health literacy in parents and young people, perceived lack of cultural competency of formal help sources, and financial costs deterring access.
Murray et al. 2010[39]	Qualitative	Birth experiences of African refugee women in Brisbane.	Brisbane, Queensland	Maternity services	Semi-structured interviews	10	Sudan (5) Liberia (1) Ethiopia (2) Somalia (2)	Refugee birthing experiences faced some unique challenges such as language barriers, the refugee experience, female genital mutilation, and healthcare staff with little cultural competence.
Neale et al. 2007[40]	Mixed Methods	Health service use and barriers of recently arrived immigrants from the Horn of Africa in Melbourne.	Melbourne, Victoria	All services	Semi-structured questionnaires	126^a^	Somalia (67) Eritrea (29) Ethiopia (24) Sudan (6)	Difficulties with language, including the availability of interpreters, were identified as the main barriers to accessing appropriate health services. Half reported difficulties with accessing services.
Nicol et al. 2014[41]	Qualitative	Refugee experience, access and understanding relating to early oral health.	Western Australia	Child dental health services	Focus groups (interviews were only with service providers)	39	Burma (16) Iraq, Kuwait (9) Sudan (5) Afghanistan (3) Burundi (2) Congo (2) Rwanda (1) Nigeria (1)	Many participants felt overwhelmed due to misinformation and low health literacy. Themes involved included parents’ past experience, resettlement issues, and enablers and barriers to accessing dental services.
Niner et al. 2013[42]	Qualitative	Experiences of pregnancy and birth before and after resettlement for Karen women in Australia.	Not specified	Maternity services	Interviews	15	Myanmar (15)	Many patients used self-reliance when emotionally distressed. They were unsettled by the medicalisation of birthing and reaction to health service varied from gratitude to feelings of discrimination.
O’Callaghan et al. 2007[43]	Qualitative	Experiences of older Vietnamese women in using medications in Fairfield, NSW.	Fairfield, New South Wales	Primary care	Semi-structured interviews (20) and focus groups (20)	40	Vietnam (40)	Women’s health literacy influence medication use and their rationale. Refugees have concerns about health professionals not favouring traditional medicine use.
Omeri, A et al. 2006[44]	Qualitative	Beliefs, practices and experiences of Afghan people in accessing healthcare in New South Wales.	New South Wales (most likely Sydney)	All services	Semi-structured interviews and focus groups	38	Afghanistan (38)	Four main themes emerge from Afghan refugee experiences of accessing healthcare: emotional responses to trauma and migration, culture specific health maintenance strategies, cultural views on mental health, barriers impeding accessibility and cultural factors influencing outcomes.
Owens et al. 2016[45]	Qualitative	Refugee and migrant women’s perceptions of using antenatal healthcare services in Perth.	Perth, Western Australia	Community-based maternity services	Semi-structured interviews	12^a^	Indonesia (1) Pakistan (1) Vietnam (1) Iran (1) Sudan (1) Myanmar (6) Thailand (1)	Women noted lack of social support during pregnancy, language difficulties, and cultural differences. They were happy with the completeness of care throughout pregnancy.
Phillips 2013[46]	Qualitative	Readily accessible remote telephone interpreting in the resettlement experience of refugees.	Not specified	Remote translation and interpreting services	Chart audit (265) and interviews (8)	273	Afghanistan, Bosnia, Burma, Iran, Iraq, Sierra Leone, Sudan	Remote interpreters do not provide the same care and attention as an on-site interpreter. Longer conversations, more interruptions, can occur with remote interpreters.
Riggs et al. 2012[47]	Qualitative	To explore the utilisation and experience of maternal and child health services in Melbourne for parents of refugee background from the perspective of users and providers.	Wyndham and Hume in Melbourne, Victoria	Maternal and child health services	Focus groups (interviews were only with service providers)	87	Karen, Iraqi, Assyrian Chaldean, Lebanese, South Sudanese, Bhutanese	Barriers for patients included access to transport, lack of confidence in speaking English. Continuity of care was appreciated and preferred. 4 identified themes included facilitating access to maternal and child health services, promoting continued engagement with the MCH service, language challenges, and what is working well and could be done better.
Riggs et al. 2016 [59]	Qualitative	Experiences of barriers, knowledge and surrounding beliefs of maternal oral health from refugees and service providers.	South-East region of Melbourne	Maternal dental health services	Focus groups (interviews were only with service providers)	27	Afghanistan (14) Sri-Lanka (13)	Afghan men provided transport, translation and their role in caring for their wives challenged traditional preconceptions. Afghan men would like health professionals to enquire about their health concerns.
Riggs et al. 2017[49]	Qualitative	Experiences of Karen women in group pregnancy care in Melbourne.	Melbourne, Victoria	Maternity services	Focus groups	19	Karen (19)	Women felt empowered and reassured when learning about pregnancy, sharing stories and developing trusting relationships in a group setting. Communication and privacy were issues encountered in the hospital.
Riggs, Yelland, Szwarc et al. 2016[48]	Qualitative	The experiences of Afghan women and men of refugee background having a baby in Melbourne, Australia.	Greater Dandenong and Casey in Melbourne	Maternal and child health services	Interviews (focus groups were only with service providers)	30	Afghanistan (30)	Afghan men found their role as a father changed in Australia but were generally pleased with the changes. Men appreciated when health professionals took an interest in them and would prefer if HCWs were responsive to issues surrounding settlement in a new country.
Robards et al. 2019 [60]	Qualitative	Understanding health system navigation and the role of technology for young people belonging to one or more marginalised groups.	New South Wales	All services	Semi-structured interviews	9^a^		Marginalised young people are ambivalent about their healthcare journey. For refugees, confidentiality concerns, discrimination and confusion over the complexity of the health system were commonly encountered themes.
Russo et al. 2015[50]	Qualitative	Emotional and social wellbeing of new mothers from Afghanistan living in Melbourne.	City of Greater Dandenong and City of Casey, Victoria	Maternal and child health services	Focus groups (28) and in-depth interviews (10)	38	Afghanistan (38)	The majority of patients reported positive experiences with HCWs and the health system They felt respected and included in the decisions regarding their care. Some discussed how their care conflicted with traditional cultural practices. Emotional challenges and changes to improve emotional wellbeing were also identified.
Samuel et al. 2017[20]	Qualitative	Narratives of health-seeking behaviours of Sri-Lankan Tamil refugees in Melbourne.	Melbourne, Victoria	All services	Semi-structured interviews	12	Sri Lankan (12)	Tamil refugees describe their health-seeking influenced by the search for the ‘good life’ that was lost or never experienced, seeking help from familiar channels in an unfamiliar context, and the desire for financial and occupational independence.
Sheikh et al. 2011[51]	Mixed methods	Identifying issues affecting newly arrived refugees in accessing an emergency department.	Liverpool Hospital, New South Wales	Emergency department	Semi-structured questionnaires	155	Africa (106) Middle East (49)	Newly arrived refugees were aware of how to call for emergency medical help, but a large proportion noted they were fearful to make phone calls due to security implications on the basis of previous experiences in their home country.
Sievert et al. 2018[52]	Mixed methods	To characterise and identify health literacy of chronic hepatitis B and barriers accessing healthcare in at-risk migrant populations.	Monash Health liver and refugee clinics in Melbourne suburbs	Liver and refugee clinics	Surveys (14) and semi-structured interviews (19)	33^a^	Afghanistan (11) Myanmar (8) South Sudan (14)	Refugees and asylum seekers living with chronic hepatitis B have competing social pressures which impact their prioritisation of health. Poor knowledge about disease, testing, services as well as language barriers and cultural differences encountered impacted accessibility of services.
Stapleton et al. 2013[53]	Mixed methods	Women from refugee background’s experiences of antenatal healthcare at an Australian tertiary public hospital.	Not specified	Maternity services	Focus groups (18), surveys (42) and chart audit (190)	250	Africa, Middle East and other countries	Patients noted differences between their traditional birthing practices and Western practices. Continuity of care throughout antenatal period provided security and support to negotiate an unfamiliar setting.
Sypek et al. 2008[54]	Qualitative	Impact of regional resettlement of refugees on rural health services and critical health infrastructure in four rural towns in NSW.	Four rural communities in New South Wales	Primary care	Interviews	7	East and West Africa Europe Middle East	Availability of appropriate primary health care services, language accessibility and mismatch in service delivery expectations were all concerns identified in health services and health infrastructure in rural NSW towns.
Valibhoy, Kaplan, et al. 2017[55]	Qualitative	Experiences of young people in using mental health services in Australia.	Mainly in Melbourne, but not specified	Mental health services	Semi-structured interviews	16	Iraq (5) Afghanistan (3) Iran (2) Sudan (1) Pakistan (1) Tanzania (1) Ethiopia (1) Côte d’Ivoire (1) DR Congo (1)	Young refugee users of mental health services describe their experiences under the themes of accessible and responsive services, cultural sensitivity, recognising the impact of psychosocial stress, appropriate treatment strategies and the therapeutic relationship.
Valibhoy, Szwarc, et al. 2017[19]	Qualitative	Description of barriers young people face in accessing mental health services in Australia.	Mainly in Melbourne, but not specified	Mental health services	Semi-structured interviews	16	Iraq (5) Afghanistan (3) Iran (2) Sudan (1) Pakistan (1) Tanzania (1) Ethiopia (1) Côte d’Ivoire (1) DR Congo (1)	Refugees face unfamiliarity with existence of services or thresholds needed to enter a service and stigma. Refugee youth more likely to turn to informal help than professional help. Negative expectations about seeking help, need for autonomy and structural barriers faced by refugees.
Wohler et al. 2017[56]	Systematic review	Systematic review into barriers culturally and linguistically diverse women face in accessing mental health services in Australia.		Mental health services				When accessing mental health services in Australia, culturally and linguistically diverse women (including refugees) face language and communication barriers, logistical barriers, barriers of cultural dissonance and have a preference for alternative interventions.
Yelland et al. 2014[57]	Qualitative	Responsiveness of health services to the social and mental health of Afghan women and men at the time of having a baby.	City of Greater Dandenong and City of Casey, Victoria	Maternal and child health services	Semi-structured interviews (focus groups were only with service providers)	30	Afghanistan (30)	Participants stated they were not asked about social circumstances despite social hardship during the antenatal and postnatal period.
Yelland et al. 2016[58]	Qualitative	Afghan refugee and service providers’ experiences of language support during pregnancy check-ups, labour and birth.	Victoria	Maternity services	Interviews (focus groups were only with service providers)	30	Afghanistan (30)	There was a lack of use of interpreters with family members often interpreting.

#### Methods and study design

Studies selected in this review were published between 2006 and 2019. Twenty-two studies used semi-structured or in-depth interviews [19, 20, 29, 30, 34, 35, 37–39, 42–46, 50, 52, 54, 55, 57–60]. Focus groups with refugees were used in 12 studies [28, 31, 33, 38, 41, 43, 44, 47–50, 53] and only 11 studies used a combination of methods to collect data [30, 32, 36–38, 43, 44, 46, 50, 52, 53]. Surveys or questionnaires were used in seven studies [32, 36, 37, 40, 51–53]. Chart audits were used in four studies [32, 36, 46, 53]. One study used field observations as part of their methodology [30]. One study was a systematic review [56].

#### Study settings

Majority of studies were carried out in Australian capital cities. Half of all the primary studies were performed in Melbourne [18–20, 29, 35–38, 40, 47–50, 52, 55, 57, 59]. Three studies did not specify the study setting [42, 46, 53]. Only one study was performed in a rural or regional area [54].

#### Participants

The perceptions of approximately 1855 refugees were captured. Five studies had a mix of refugees and migrants and did not identify those with refugee status [33, 35, 38, 40, 45, 52]. One study had a mix of refugees and other marginalised groups [60]. Furthermore, some studies appear to have published different findings from the same data set [19, 55, 58, 59].

#### Country of origin

Refugees came from over 39 countries or regions. The most investigated country of origin was Afghanistan; others were the African continent, countries from the Middle East, Vietnam, Sri Lanka, and South-East Asia.

#### Health services explored

A diverse range of services were captured. Maternity services were the most frequently investigated [29, 32, 35, 39, 42, 47–50, 53, 57–59]. Six studies investigated all services [20, 33, 37, 40, 44, 60] which was followed by mental health [19, 38, 55, 56] and primary care [30, 43, 54].

### Risk of bias within studies

The results of the qualitative appraisal are summarised in Table 2. Quantitative, mixed methods, and systematic review appraisals are summarised in Table 3. Seven articles were of high quality, 9 articles were of acceptable quality and 19 articles were low quality. For all qualitative and mixed methods studies, theoretical appraisal was omitted from assessment as only 8 articles stated their methodology and paradigm [20, 35, 38, 39, 42, 45, 50, 59].

**Table 2:** Appraisal of Qualitative Articles using the Modified Critical Appraisal Tool (MCAT). Theoretical appraisal was omitted from assessment as only 8 articles clearly stated their methodology and paradigm [20, 35, 38, 39, 42, 45, 50, 59].

Study	Design	Fundamentals	Credibility	Dependability	Reporting	Utility	Overall Quality
Robards et al. 2019 [60]	Qualitative	Satisfied	Satisfied	Satisfied with reservations	Satisfied with reservations	Low utility	High quality
Bellamy et al. 2017[28]	Qualitative	Satisfied	Satisfied with reservations	Satisfied with reservations	Satisfied	Moderate utility	High quality
Owens et al. 2016[45]	Qualitative	Satisfied	Satisfied	Satisfied with reservations	Satisfied	Moderate utility	High quality
McCann et al. 2016[38]	Qualitative	Satisfied	Satisfied with reservations	Satisfied	Satisfied	Mild utility	High quality
Russo et al. 2015[50]	Qualitative	Satisfied	Satisfied with reservations	Satisfied	Satisfied	Mild utility	High quality
Murray et al. 2010[39]	Qualitative	Satisfied with reservations	Satisfied	Satisfied	Satisfied	Moderate utility	High quality
Liamputtong et al. 2006[35]	Qualitative	Satisfied	Satisfied with reservations	Satisfied with reservations	Satisfied	Low utility	High quality
Samuel et al. 2017[20]	Qualitative	Satisfied	Satisfied with reservations	Satisfied with reservations	Satisfied with reservations	Moderate utility	Acceptable quality
McBride et al. 2017[37]	Mixed Methods	Satisfied	Satisfied with reservations	Satisfied with reservations	Limitations	Mild utility	Acceptable quality
Yelland et al. 2016[58]	Qualitative	Satisfied	Satisfied with reservations	Satisfied with reservations	Limitations	Low utility	Acceptable quality
Cheng et al. 2015[18]	Qualitative	Satisfied with reservations	Limitations	Satisfied	Satisfied with reservations	Mild utility	Acceptable quality
Nicol et al. 2014[41]	Qualitative	Satisfied	Satisfied with reservations	Satisfied with reservation	Satisfied with reservations	Moderate utility	Acceptable quality
Riggs et al. 2012[47]	Qualitative	Satisfied	Satisfied with reservations	Satisfied with reservations	Satisfied with reservations	Moderate utility	Acceptable quality
Carolan et al. 2010[29]	Qualitative	Satisfied	Satisfied with reservations	Severe Limitations	Satisfied	Moderate utility	Acceptable quality
O’Callaghan et al. 2007[43]	Qualitative	Satisfied with reservations	Limitations	Satisfied	Satisfied with reservations	Mild utility	Acceptable quality
Omeri, A et al. 2006[44]	Qualitative	Satisfied	Satisfied with reservations	Satisfied	Limitations	Mild utility	Acceptable quality
Sievert et al. 2018[52]	Mixed Methods	Satisfied	Limitations	Limitations	Limitations	Moderate utility	Low quality
Valibhoy, Szwarc, et al. 2017[19]	Qualitative	Satisfied	Severe Limitations	Severe Limitations	Limitations	Moderate utility	Low quality
Valibhoy, Kaplan, et al. 2017[55]	Qualitative	Satisfied	Severe Limitations	Severe Limitations	Satisfied with reservations	Moderate utility	Low quality
Riggs et al. 2017[49]	Qualitative	Satisfied	Satisfied with reservations	Limitations	Limitations	Mild utility	Low quality
Riggs et al. 2016[48]	Qualitative	Satisfied	Limitations	Satisfied with reservations	Satisfied with reservations	Moderate utility	Low quality
Riggs, Yelland, Szwarc et al. 2016[59]	Qualitative	Satisfied	Limitations	Limitations	Satisfied with reservations	Mild utility	Low quality
McBride et al. 2016[36]	Mixed methods	Satisfied	Limitations	Satisfied with reservations	Limitations	Moderate utility	Low quality
Kay et al. 2016[34]	Qualitative	Satisfied	Limitations	Severe Limitations	Limitations	Mild utility	Low quality
Yelland et al. 2014[57]	Qualitative	Satisfied	Limitations	Limitations	Satisfied with reservations	Mild utility	Low quality
Clark et al. 2014[31]	Qualitative	Satisfied	Limitations	Severe Limitations	Limitations	High utility	Low quality
Stapleton et al. 2013[53]	Mixed methods	Satisfied	Limitations	Satisfied	Limitations	Mild utility	Low quality
Phillips et al. 2013[46]	Qualitative	Limitations	Severe Limitations	Satisfied with reservations	Severe Limitations	Mild utility	Low quality
Niner et al. 2013[42]	Qualitative	Limitations	Severe Limitations	Severe Limitations	Limitations	Moderate utility	Low quality
Correa-Velez et al. 2012[32]	Mixed methods	Satisfied	Limitations	Limitations	Satisfied with reservations	Mild utility	Low quality
Sheikh et al. 2011[51]	Mixed methods	Limitations	Severe Limitations	Severe Limitations	Limitations	Mild utility	Low quality
Henderson et al. 2011[33]	Qualitative	Satisfied	Limitations	Satisfied with reservations	Satisfied with reservations	Mild utility	Low quality
Sypek et al. 2008[54]	Qualitative	Satisfied	Limitations	Satisfied with reservations	Severe Limitations	High utility	Low quality
Neale et al. 2007[40]	Mixed Methods	Satisfied with reservations	Limitations	Satisfied with reservations	Limitations	Low utility	Low quality

**Table 3:** Quantitative and Mixed Methods Appraisals

Study	Study Design	Tool Used	MCAT Appraisal	Final Appraisal
McBride et al. 2017[36]	Mixed Methods	MMAT	Acceptable quality	Acceptable quality
Sievert et al. 2018[52]	Mixed Methods	MMAT	Low quality	Low quality
Wohler et al. 2017[56]	Systematic review	JBI	Not applicable	Low quality
Neale et al. 2007[40]	Mixed Methods	MMAT	Low quality	Low quality
McBride et al. 2016[36]	Mixed Methods	MMAT	Low quality	Low quality
Stapleton et al. 2013[53]	Mixed Methods	MMAT	Low quality	Low quality
Correa-Velez et al. 2012[32]	Mixed Methods	MMAT	Low quality	Low quality
Sheikh et al. 2011[51]	Mixed Methods	MMAT	Low quality	Low quality

### Synthesis of results

Three major concepts that are inter-related emerged from the review: personal engagement, service and system issues with access, and trust and privacy.

#### Engagement: refugees’ struggle to engage with health services

As the Centre for Advancing Health defines, engagement is the actions individuals must take to obtain the greatest benefit from the healthcare services available to them [61]. The struggle that refugees experience to engage with healthcare services in Australia was evident. Refugees must take certain actions to negotiate care, which can be conceptualised as a three-step process.

##### Refugees are in an unfamiliar environment

Refugees perceive many differences in the Australian healthcare system including language, health system, and culture. This unfamiliarity hinders their engagement with services.

Differences in language results in challenges in the clinical setting [19, 28, 30–35, 37, 39–42, 44–47, 51–54, 56, 58, 59] which are perpetuated by a lack of use of interpreters [28, 30–34, 39–42, 44–49, 52, 56–58]. This was either because refugees were unaware of their availability [28, 39], there were limited or no interpreters available [40, 44, 52], interpreters were substituted by family members [30, 31, 39, 45, 48, 58], refugees felt reluctant to use interpreters [56], or felt that it was not in their right to ask for one [58]. Quite often, language barriers resulted in poor understanding [39, 51].

*“When you don’t speak the language, you lack a lot of things.” Participants indicated that often they only partially understood what health care providers said, or they did not understand at all* [39].

In part, due to language issues, refugees had poor health literacy, which is defined as the capacity to obtain, process and understand basic health information and services to exercise their agency [62]. This was displayed through poor understanding in areas of medical interventions, health, disease, and the health system [19, 29, 31–34, 38–42, 44, 47, 48, 52, 53, 56, 59].

*“I don’t see the point to look inside with the machine (ultrasound). Maybe it will kill the child.”* [29]

Healthcare differences between the country of origin and Australia also contributed to a lack of understanding, concern, isolation, or distress [29, 39, 44].

*“In the village in Africa when you are having a baby you are sitting down like this (motions squatting). Yeah, but here it is very different. You sleep (lie in bed), and that is make her scared.”* [39]

Refugees also held different expectations about the Australian healthcare system [28, 33].

*There was an expectation that a visit to a doctor would involve an injection, such as they experienced in Sudan (e.g. antibiotics and malaria injections). There was a sense among the group that if an injection was not given, then the GP had not satisfactorily dealt with their health issue* [33].

Cultural differences between refugees and healthcare staff also played a role in creating an unfamiliar environment. Refugees often expressed concerns regarding the cultural incompatibility of services or the inability to observe cultural practices [29, 32, 34–36, 38, 42, 44, 45, 48, 50, 52, 53, 55, 57]. A lack of cultural sensitivity was also experienced [29, 32–35, 42, 44, 56]. Some cultural values that refugees reported were different related to the importance of family support, discipline of children, and the care of the elderly [44].

*“…they did not wish to risk being admitted to hospital, because they would be separated from their family and would miss their own cultural food*.” [33]

Refugees reported that some healthcare staff did not understand their past trauma, gave insensitive advice, or caused distress by reminding them of their past [55]. Healthcare staff often probed problems or sensitive issues refugees regarded were inappropriate to discuss in their culture or religion [19, 55].

Some healthcare staff did not try to understand their cultural backgrounds or needs [40, 44] and at times, making incorrect assumptions [55]. In addition to this, refugees felt rejected when healthcare staff were dismissive of their cultural or traditional health practices instead of respectfully considering its legitimacy [33, 43, 50].

*“I felt like I was judged by my doctor… I wanted to do things according to my tradition, but I was expected to do things differently...”* [50]

It is therefore not surprising that refugees often preferred health practitioners who were of their same background with good understanding of refugee and cultural issues [32, 33, 38, 51, 55].

##### The importance of health information sharing

The importance of providing and understanding health information needs of refugees was captured through the concept of information sharing. To ameliorate the unfamiliarity and misunderstanding, refugees require high quality information sharing practices to help them navigate a complex health system to overcome difficulty [28, 33, 39, 40, 44]. Information about the availability of services was scarce or insufficient [40, 56, 59].

*“We don’t know where everything is (health services) ... nobody knows. Sometimes it is word-of-mouth...”* [33]

Health information was often culturally inappropriate, not translated, or targeted [32–34, 40, 41, 44, 47, 48, 59]. For example, refugees valued practical information over medical information [45, 53]. In some instances, refugees were provided with incorrect information, or received mixed messages [34, 41, 42, 59].

*“My family was sent home... I had birth by baby been pulled by machine... nobody informed me as to what happened... my family are not happy about it.”* [32]

In contrast, some refugees were overloaded with information during the settlement process [33].

*“Maybe they can explain [the health system] to us, but when we just arrived we’ve got so many things to do so we got overloaded – we couldn’t remember.”* [33]

Furthermore, refugees felt that healthcare staff were challenged by time constraints and commonly attributed this as a barrier to information sharing [45, 53, 59].

*“The GP and the maternal child nurse, if you not ask them they not giving you information because of limit of time. It’s hard for them to tell us.”* [59]

Refugees regarded information sharing as important [29, 32, 33, 38, 39, 47, 50] as having control of information and the ability to ask questions promoted power, autonomy and confidence [38, 39, 45, 49].

*Contrary to the experience of “not knowing” was a sense of deeply valuing information when it was available. Where information was understood by participants, they felt more in control, relaxed, and comfortable* [39].

*For women with little social support, being informed about their pregnancy and able to ask questions may contribute to them feeling empowered, and a positive perception of their pregnancy* [45].

On the contrary, a lack of information or understanding resulted in disempowerment, distress, and fear [33, 39, 42, 51]. Some refugees were not provided with adequate information or explanation about their condition, treatment, or process of care. This was often compounded by a language barrier, or a lack of use of interpreters [31–34, 39, 41, 42, 53, 59].

*During delivery, she [a Karen mother] related that it was communicated to her that her son, “did not have a head,” in reaction to which she recalls: “my heart was shaking.” The delivery was successful; although her son required intensive care, he survived and thrived… Two years later Ruth still did not fully understand why or how the medical procedures were performed; and confusion and distress over this is evident in her account* [42].

Disempowerment was particularly prominent when care was related to children of refugees. A study exploring maternal and child health services identified that refugees were cautious to question the plan of treatment, fearing that it would make them appear neglectful and result in legal repercussions [47].

##### Reclaiming power and autonomy through familiar means

When refugees were not able to overcome the challenges of information sharing, they resorted to familiar means to reclaim their power and autonomy to exercise their own agency. Family, friends, interpreters, support workers, past experiences, and own cultural knowledge were familiar avenues that were often resorted to.

Support from family promoted refugees’ engagement with health services through language, transport and navigating the health system. Husbands played an important role in these areas [45, 48–50, 53, 58–60]. Men took on new roles that were not traditionally practiced in their country of origin [44, 45, 48, 50, 53].

“*In Afghanistan I wouldn’t go to appointments with my wife... but here I can spend the time with my wife.*” [48]

Overcoming language barriers through use of family members was common [30, 31, 39, 45, 48, 58]. However, this was not without issues as family members’ English may be insufficient and privacy issues arose [39, 45, 48]. Family members, friends, and settlement workers also helped refugees navigate the health system [30, 31, 33, 37, 44, 55, 60]. Sometimes children were relied upon, at the expense of their educational commitments [33].

*“We need to go with a male or with our teenage children who will miss school and we feel bad...”* [33]

*“My daughter without her I can’t do anything, shopping, money, there is no other way we don’t know what to do, sometimes children have to be forced to help.”* [31]

While family members are often required to assist, refugees felt they were often excluded from their clinical care [32, 33, 42, 48, 49, 57]. However, reliance on family members was evident and potentially disempowering as it removed their opportunity to engage with services themselves. This was perpetuated by healthcare staff when refugees were told to bring family members to translate [37, 45, 58].

*Some women were dependent on their partners for interpreting needs, and so it could be argued it was due to necessity that husbands were present at the birth* [45].

Family and friends could equally be discouraging and act as barriers to appropriate healthcare [19, 55].

*In Majok’s experience, friends were discouraging (“someone else told me like, ‘nah, don’t go to her, she’s gonna talk a lot’ ...My friend told me, ‘don’t go to this guy, this guy maybe he’s crazy guy”’) while family acted as facilitators (“Family ... they want you badly to go ... The families know better than you, they care”)* [55].

Cultural and religious stigma perpetuated by family, friends, and religious leaders were barriers to accessing services, particularly mental health services [19, 20, 38, 56].

Use of professional interpreters enabled refugees to reclaim power and autonomy. Well-regarded interpreters helped overcome cultural and linguistic barriers [31, 34, 37, 46, 57]. However, problems with using interpreters included the lack of privacy, the wrong interpreter organised, interpreters taking longer in consultations, lack of rapport with over-the-phone interpreters, and interpreters translating incorrectly [28, 32, 33, 46, 52, 54, 56, 58, 60]. Gender preferences of the interpreter, availability, technical and appointment difficulties were other common issues [40, 44].

The use of traditional medicines was also another means for refugees to take control of their own health [33, 42, 43, 50, 52].

*Participants in all four CALD groups reported that when a family member was sick, they first tried to apply traditional medicines from their country. If this did not work, they would seek a doctor, but this was as a last resort, particularly due to the expense* [33].

#### Access: system and service issues

A predominant part of refugees’ experiences in the Australian healthcare system were barriers and enablers faced in accessing healthcare services. Access issues interacted with the way refugees engaged with services. These experiences can be conceptualised using Penchansky and Thomas’ definition of access, which is a broad concept describing the fit between the patient and the health care under the dimensions of acceptability, accommodation, accessibility, affordability, and availability [63].

##### Acceptability of services

Acceptability relates the attitudes that refugees have towards a health service as well as the attitudes that providers have to refugees [63].

Overall, refugees have a positive experience in the Australian healthcare system including gratitude for the care offered, appreciation of staff for their expertise, and positive attitudes [20, 29, 33, 37, 38, 41, 42, 45, 47–50, 53, 55, 59]. Refugees appreciated a caring connection, sensitivity, and respect of their cultural practices [29, 30, 32, 33, 37, 39, 44, 50, 54, 55]. When rapport was built or when they felt listened, this was also appreciated [47, 57]. These positive attributes of healthcare staff promoted attendance, helped with understanding, and impacted client satisfaction [29, 37].

Refugees regarded healthcare professionals as competent and were skilled in their area of expertise [29, 38, 41, 42, 47, 50, 53, 55]. However, refugees then often felt that they had to be agreeable to management plans of doctors, creating a dilemma when plans contravened traditional knowledge [35, 47, 50]. This conflict affected refugees’ perceptions on the acceptability of services.

Furthermore, these positive accounts may not be entirely representative of the real feelings of refugees. In some studies, researchers suspected that refugees may have over-reported their degree of satisfaction of their host country as a display of their satisfactory adjustment in Australia [39, 42, 47, 52, 53]. Refugees may feel beholden to the Australian system and there may also be culturally basis towards their expression of dissatisfaction [42].

Refugees, especially women, had strong gender preferences for their service providers, which were usually not met [33, 39, 40, 44, 45, 48, 50, 52, 56–58]. At times, this was a matter of cultural safety and a point of contention with staff who had different values [28, 44, 45, 50]. This impacted the degree to which refugees were able to confide with healthcare staff [57, 58].

*“The only disrespect was that we couldn’t choose the doctor to be female during labour. My wife was uncomfortable and worried. They (health professionals) said it’s no issue for us and shouldn’t be for you guys.”* [48]

When refugees were actually or perceived to be discriminated against by staff, this negatively impacted the acceptability of services [19, 42, 44, 49, 60].

*Rosy added later in her account that she did not think the doctor would have “treated white people in the same way,” identifying the treatment as discriminatory* [42].

##### Accommodation of services

Accommodation, which describes how supply services are organised to accept patients, as well as the ability for patients to accommodate to these factors and their perceived appropriateness, influenced the degree of access that refugees had with services [63].

The childrearing roles of family members, as well as the lack of childcare services, impacted negatively on access to healthcare [47, 53, 56]. Entry point barriers such as complex referral pathways and narrow eligibility criteria made it difficult for refugees to access services [19, 55]. Refugees appreciated when these barriers were removed with walk-in clinics [53]. Bureaucracy and difficulties with making appointments further affected access [30–33, 37, 45, 47, 53, 55, 59] and were compounded by language barriers [30, 31, 47, 53, 59].

##### Accessibility of services

Accessibility identifies the relationship between the location of the service and the location of the clients and how this affects their degree of access [63]. Difficulties with transport [30–32, 37, 41, 44, 45, 47, 49, 53, 56, 57, 59] and health services being too far away made transport costs an issue [19, 40, 44, 54]. Co-location of multiple services was well-regarded by refugees [30, 37, 45, 55].

##### Affordability of services

Affordability relates the cost of the service to the patient’s income and their ability to pay [63]. Refugees described costs of services and pharmaceuticals as a barrier to healthcare [19, 20, 29, 30, 33, 34, 38, 53, 54, 56, 59]. The economic impact of taking time off work to seek healthcare, which often involved other family members, cumulated into a costly exercise [53]. Assistance from family and traditional medical practices were used to avoid the costs. Professional healthcare were a last resort [33].

##### Availability of services

Availability identifies the relationship between the volume supplied and type of services made available, in relation to the volume of patient demand and types of need [63]. Access is influenced by this and refugees reported unmet health needs in rural and regional towns where there were a lack of specialist services [54].

#### Trust and privacy: influencing all aspects of access and engagement

Trust and privacy are issues that influence engagement and access. It is a pervasive issue that influences the degree of familiarity refugees can have with the health system, the amount of information that they can share with healthcare staff and the degree of power and autonomy that they can exercise.

Limited understanding of the health system impacts the trust that patients have with the services questioning the efficacy of treatments offered [20, 29, 33, 35, 42, 54]. Refugees were also cautious of the intentions of healthcare staff with concerns that they may disclose information to government agencies which would influence their visa status [39, 47, 52, 54, 56].

*“They know the appointment is going to be all talking, it’s nothing interesting”, however he thereafter commented, “maybe they scared they gonna find something wrong with them”, pointing to deeper fears* [19].

Fears about confidentiality affected the degree to which refugees were able to confide with healthcare staff [19, 38, 46, 60]. When breeches in privacy or their privacy was violated, refugees experienced a sense of distress and disempowerment [44, 49].

*In these instances, women felt their preferences were ignored. This was compounded by women’s reticence to advocate for themselves, leading them to feel voiceless. “They would ask questions and I didn’t want to answer it straight away, because I don’t feel comfortable with them… I didn’t feel comfortable to say to them ‘Why are you here?’”* [49]

Continuity of care promoted trust, avoided unnecessarily repeating histories to focus on current issues, promoted confidence, increased satisfaction [30, 32, 45–47, 49, 53, 55–57], and reduced the need to revisit past traumatic events [55].

*“The more I repeat the same thing that they ask me I get more depressed, because I’m bringing out the same thing again and again, and it’s making me more emotional. So every time I went or somebody new came I would not talk.”* [55]

## Discussion

### Summary of evidence

**Fig. 2:**
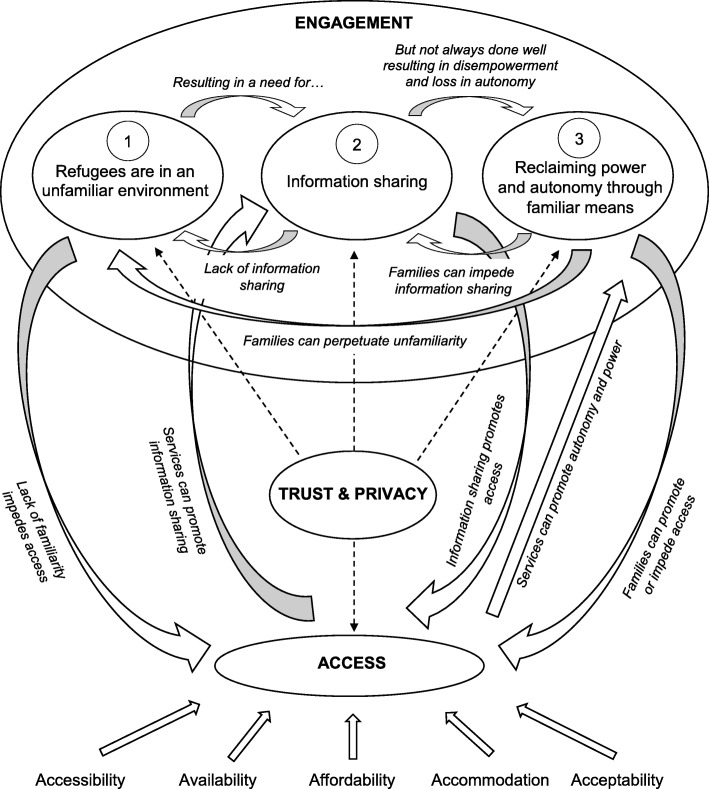
Explanatory Model for the Experiences of Refugees in Using Healthcare Services in Australia

The findings from this synthesis suggest that refugees face major barriers in their engagement and access of healthcare services in Australia which they must take action to overcome. These barriers are complex and inter-related as shown in the model used to explain the findings (Fig. 2). Refugees are in an unfamiliar environment due to perceived differences in language, culture, and health systems. This results in a need for effective information sharing to promote power and autonomy to navigate the health system. However, this is often done ineffectively, resulting in disempowerment and loss of autonomy. To exercise their agency, refugees reclaim their power and autonomy through familiar means such as family, friends and interpreters. At times, they may be successful, but reliance on family and friends may perpetuate their unfamiliarity with the health system. These factors collectively influence the degree of engagement that they have with the health system. Interacting with this, refugees face access issues which can be conceptualised using Penchansky and Thomas’ concept of access [63]. Trust and privacy ultimately affects all aspects of access and engagement. The links between access and engagement were through effective information sharing and successful reclamation of power and autonomy, which may be promoted or hindered by family or services.

The model describes a response that refugees use to make the best of their means in a foreign country (Fig. 2). It reflects the harsh realities of their circumstances, which must be negotiated to engage with health services.

The findings relating to individual barriers and enablers faced in the clinical setting is consistent with international literature. A literature review of refugee experiences of general practice involving papers from 12 resettlement countries noted prominent barriers to access, language barriers, issues with the doctor-patient relationship, and the cultural acceptability of medical care [18]. In another scoping review of refugee experiences of healthcare in nine host countries, communication and information, language barriers, access barriers, continuity of care, discrimination, cultural competency, and knowledge about the healthcare system were all raised as issues [17].

This systematic review adds to the body of evidence by providing an Australian context of the health system. The findings emphasise the importance of information sharing and noting the familiar avenues that refugees resort to maintain power and autonomy. It clusters their experiences into the dynamic categories of engagement, access, and trust and privacy. This has not been previously captured as an interacting process and may be an oversight of previous literature which was captured in the present thematic synthesis and systematic review. An explanatory model is presented to explain how refugees negotiate a complex health system (Fig. 2). These findings may be transferrable to other settings as previous systematic reviews of refugee experiences in general practice and scoping reviews of refugee experiences in their host countries identified aspects of these interacting processes [17, 18].

Overall, the evidence supporting these review findings is highly robust with high confidence in four review findings and moderate confidence in one review finding, as assessed by the GRADE-CERQual approach (Table 4). Despite the high number of low-quality studies, findings were coherent and adequate across studies. There were a satisfactory number of high-quality studies supporting each review finding.

**Table 4:** GRADE-CERQual Evidence Profile (EP)

Summary of Review Finding	Studies contributing to the review finding	Methodological Limitations	Coherence	Adequacy	Relevance	CERQual Assessment of Confidence in the Evidence	Explanation of CERQual Assessment
1. Refugees are in an unfamiliar environment, manifested by differences in culture, differences in language, and differences in health systems	[19, 28–59]	Moderate concerns: A total of 6 articles were of high quality, 8 articles were of acceptable quality, and 19 articles were of low quality.	Minor concerns: data reasonably consistent across studies, with some minor deviations	No or very minor concerns: explanatory finding: very high data quantity and richness	No or very minor concerns	High confidence: It is highly likely that the review finding is a reasonable representation of the phenomenon of interest	Although there were moderate concerns over the methodology of some low-quality articles, we have high confidence that the coherence, adequacy and relevance of the data represents the phenomenon well.
2. Information sharing promotes power and autonomy and is important to help refugees navigate a complex health system. A lack of information sharing results in disempowerment and loss in autonomy.	[28, 29, 31–34, 38–42, 44, 45, 47–51, 53, 56, 59]	Moderate concerns: A total of 5 articles were high quality, 4 articles were acceptable quality, and 12 articles were low quality.	Minor concerns: data reasonably consistent across studies, with some minor deviations	No or very minor concerns: explanatory finding: very high data quantity and richness	No or very minor concerns	High confidence: It is highly likely that the review finding is a reasonable representation of the phenomenon of interest	Although there were moderate concerns over the methodology of some low-quality articles, we have high confidence that the coherence, adequacy and relevance of the data represents the phenomenon well.
3. Refugees reclaim power and autonomy through familiar means. At times, they may be successful, but this may perpetuate their unfamiliarity with the health system	[19, 20, 28, 30–34, 37–40, 42–46, 48–50, 52–60]	Moderate concerns: A total of 6 articles were high quality, 6 articles were acceptable quality, and 17 articles were low quality.	Minor concerns: data reasonably consistent across studies, with some minor deviations	No or very minor concerns: explanatory finding: very high data quantity and richness	No or very minor concerns	High confidence: It is highly likely that the review finding is a reasonable representation of the phenomenon of interest	Although there were moderate concerns over the methodology of some low-quality articles, we have high confidence that the coherence, adequacy and relevance of the data represents the phenomenon well.
4. Refugees face barriers and enablers in accessing health services which are related to the acceptability, accommodation, accessibility, affordability, and the availability of health services	[19, 20, 28–35, 37–42, 44–50, 52–60]	Moderate concerns: A total of 6 articles were high quality, 8 articles were acceptable quality, and 17 articles were low quality.	Minor concerns: data reasonably consistent across studies, with some minor deviations	No or very minor concern: descriptive finding, high quantity in data	No or very minor concerns	High confidence: It is highly likely that the review finding is a reasonable representation of the phenomenon of interest	Although there were moderate concerns over the methodology of some low-quality articles, we have high confidence that the coherence, adequacy and relevance of the data represents the phenomenon well.
5. Trust and privacy influence all aspects of access and engagement	[19, 20, 29, 33–35, 38, 39, 42, 44, 46, 47, 49, 52–54, 56, 60]	Moderate concerns: A total of 4 articles were high quality, 4 articles were acceptable quality, and 10 articles were low quality.	Minor concerns: data reasonably consistent across studies, with some minor deviations	Moderate concerns: descriptive finding: data quantity not sound, but considerably rich	No or very minor concerns	Moderate confidence: It is likely that the review finding is a reasonable representation of the phenomenon of interest	There were moderate concerns over methodology of articles and the adequacy of the data.

The authors are not confident that the review findings represent Australia as a whole. Half of all the primary studies were performed in Melbourne. Research is largely concentrated in metropolitan areas of Victoria, New South Wales, and South-East Queensland. Only one study investigated rural or regional areas [54]. There currently exists a gap in the available literature in rural and regional areas. Further research is needed in these settings, particularly in Northern Australia (Northern Territory and North Queensland), as refugee services and their community contexts will be considerably different compared to their metropolitan counterparts.

Almost all the included studies did not report reflexivity, research paradigm, or theoretical perspective. This risks inaccurate interpretation of the data by third parties or potential biases authors may have onto their own interpretations and methodologies [64]. As such, this affected the credibility of articles and was reflected in their appraisal (Table 2).

### Implications

The evidence arising from the experiences of refugees suggests that clinicians need to be more aware of providing language support, consideration of their biopsychosocial contexts, sensitivity, and own attitudes. Clinicians need to identify which patients are refugees and when professional interpreters should be used. It is the policy of most States and Territories to provide interpreters for those who have difficulty with English [11, 65–67]. However, these policies do not extend into private practice. Interpreters are used in less than 1 in 100 Medicare-funded consultations despite 1 in 35 Australians having poor English [68]. Research examining effective means to promote use of interpreters in the clinical setting is needed.

For health administrators, training of healthcare staff as part of continuing professional development is essential to promote cultural competency and sensitivity. A systematic review examining interventions improving cultural competency noted that training had positive impacts on provider outcomes [69]. However, the study had difficulty determining which types of training had the best outcomes. Further research is required in this area to examine effective means of delivering cultural competency training.

Health educators also play a role in influencing the cultural competency and sensitivity of future healthcare staff. Different models of delivering this education have been used overseas [70, 71]. Education on refugee health in an Australian context will need to prepare graduates with cultural sensitivity and confidence in approaching patients of refugee backgrounds. This includes reflective skills to understand their pre-existing prejudices and attitudes, as well as empathy for the refugee context. Further research is required to establish best method of delivering this curriculum in an Australian setting.

For policy makers, promoting refugee health nurses or bilingual support staff can help refugees navigate the health system. Research evaluating the role of refugee health nurses has shown success in providing clinical support, advocacy, and education [36]. However, their roles are challenged by the workload, communication, and tension between services [72]. Their roles can range from assisting refugees to navigate the health system, carrying out cultural sensitive assessments, to improving the clinical capacity of health services. Upscaling their availability in the community to ensure greater access should be a priority.

Incentives that allow general practitioners adequate time to provide health information to refugees may be another viable avenue to overcome information sharing barriers. Interviews involving general practitioners providing care to refugees identified remuneration as a barrier [73].

Specialist refugee clinics that offer multiple services and on-site interpreting may be a possible solution to overcome barriers that refugees face. However, removing refugees out from primary care into specialist clinics may pose challenges such as reliance and follow-up issues. Although some specialist refugee clinics already operate in New South Wales and Victoria, further research investigating models of care is necessary [74].

The novel finding in our review identified that refugee experiences can be considered under the broad dynamic categories of engagement, access, and trust and privacy which has not been previously captured as an interacting process. Further research is required to examine the transferability of the present review findings in an international context. The authors believe that this may be possible as past reviews have identified aspects of the present model [17, 18].

This review did not capture the experiences of asylum seekers and health service providers which is integral to understanding the broader experience of this population group. All refugees would have previously been asylum seekers and data from this population group may be able to inform the early settlement health needs of this vulnerable population. Data from health service providers would be able to provide another perspective on the needs and concerns of refugees, as well as the quality of care, how care can be more appropriately delivered, and how refugees are able to negotiate their care [75]. The research team is currently undertaking a systematic review on the experiences of Australian healthcare staff working in refugee health which can potentially complement this study.

### Limitations

Synthesis of qualitative data removes the data from its context and generalises the results into different contexts. It risks inaccurately representing or interpreting the data from the original research [21, 76]. Close adherence to interpretations of the original authors as well as making available the aims, settings, methods and sample characteristics of each study (Table 1) allows readers to judge for themselves whether or not the contexts of the studies reviewed are similar to their own [21].

The reporting restrictions of journals may have affected adequate assessment of the methodological credibility of articles. This unfairly disadvantages qualitative researchers from adequately reporting their methodological rigour. However, the PRISMA Explanation and Elaboration notes that this should not be an excuse for omission [22]. As such, most articles were of low quality when appraised as strict adherence to the appraisal tools were made. Articles were not provided the benefit of the doubt.

The review was unable to assess potential dissemination bias in the studies included. Although it is likely to be a prominent issue in qualitative studies, there are no effective means of assessing this. There is a possibility of dissemination bias in four studies that are likely to have drawn findings from the one data set but had different reported findings and interpretations. However, there is uncertainty if this is the case [19, 55, 58, 59]. Further methodological research is required in this area and the authors acknowledge current projects undertaken by GRADE-CERQual [77].

One limitation of this study may have been related to the inclusion criteria of studies. Studies that had explicit mention of refugees even if they belonged to a heterogenous group involving non-refugees (e.g. immigrants and skilled workers) were included for analysis. Although the authors made every effort to identify the relevant findings that pertained to refugees, some studies did not clearly delineate their population characteristics which made it difficult for the assessor to adequately consider the data [33, 35, 38, 40, 45]. These studies were included in the review analysis but were subject to lower levels of confidence when it was appraised. For systematic reviews, the primary papers were referred to ensure that findings related to refugee populations [56].

## Conclusions

Through the synthesis of literature documenting the experiences of refugees in the Australian healthcare system, the major concepts of engagement, access, and trust and privacy encapsulated their narratives. The access barriers identified were largely consistent with other literature. However, this study emphasised the importance of information sharing, and noting the familiar avenues that refugees resort to maintain power and autonomy. This has not been previously captured as an interacting process. However, there continues to be a lack of available data from rural and regional areas and further research is needed in these settings which are vastly different to metropolitan areas. Implications of this study can be applied to clinical practice, health administration, health education, and health policy, by addressing service provider attitudes and the availability of services. Further research is required to examine how these recommendations can be delivered effectively.

## Additional files

Additional file 1: Search Strategy. Search strategy used in Scopus and MEDLINE. (DOCX 23 kb)

Additional file 2: Modified Critical Appraisal Tool (MCAT). A standardised qualitative appraisal tool used by authors to appraise studies with qualitative data. (DOCX 26 kb)

## Data Availability

All data generated or analysed during this study are included in this published article.

## Abbreviations

ENTREQ: Enhancing transparency in reporting the synthesis of qualitative research
GRADE-CERQual: Confidence in the Evidence from Reviews of Qualitative Research
JBI: Joanna Briggs Institute
MCAT: Modified critical appraisal tool
MMAT: Mixed methods appraisal tool
PRISMA: Preferred reporting items for systematic reviews and meta-analyses

## References

[1] Shawyer F, Enticott JC, Block AA, Cheng IH, Meadows GN (2017). The mental health status of refugees and asylum seekers attending a refugee health clinic including comparisons with a matched sample of Australian-born residents. BMC Psychiatry..

[2] Johnston V, Smith L, Roydhouse H (2012). The health of newly arrived refugees to the top end of Australia: results of a clinical audit at the Darwin refugee health service. Aust J Prim Health..

[3] Masters PJ, Lanfranco PJ, Sneath E, Wade AJ, Huffam S, Pollard J, Standish J, McCloskey K, Athan E, O'Brien DP (2018). Health issues of refugees attending an infectious disease refugee health clinic in a regional Australian hospital. Aust J Gen Pract.

[4] Hynie M (2018). The social determinants of refugee mental health in the post-migration context: a critical review. Can J Psychiatr.

[5] Annual Report 2016-2017. Department of Immigration and Border Protection, 2017. https://www.homeaffairs.gov.au/reports-and-pubs/Annualreports/2016-17/Complete.pdf. Accessed 14 Aug 2018.

[6] Coffey GJ, Kaplan I, Sampson RC, Tucci MM (2010). The meaning and mental health consequences of long-term immigration detention for people seeking asylum. Soc Sci Med.

[7] Newman L, Proctor N, Dudley M (2013). Seeking asylum in Australia: immigration detention, human rights and mental health care. Australas Psychiatry.

[8] Newman LK, Procter NG, Dudley M (2011). Suicide and self-harm in immigration detention. Med J Aust.

[9] Killedar A, Harris P (2017). Australia's refugee policies and their health impact: a review of the evidence and recommendations for the Australian government. Aust N Z J Public Health.

[10] Northern Territory Refugee Vaccination Policy. Northern Territory Government, 2011. https://digitallibrary.health.nt.gov.au/prodjspui/bitstream/10137/974/1/Northern%20Territory%20Refugee%20Vaccination%20Policy.pdf. Accessed 14 Aug 2018.

[11] Refugee health and wellbeing: a policy and action plan for Queensland 2017–2020. Brisbane: State of Queensland (Queensland Health), Brisbane. 2017. https://www.health.qld.gov.au/__data/assets/pdf_file/0031/646078/refugee-policy.pdf. Accessed 14 Aug 2018.

[12] The Victorian refugee and asylum seeker health action plan 2014–2018. State of Victoria, Department of Health, 2014. https://www2.health.vic.gov.au/Api/downloadmedia/%7B6E6F8723-0369-4DA0-B504-59397A81A679%7D. Accessed 14 Aug 2018.

[13] MBS Health Assessments Items 701, 703, 705, 707 and 715 In: Medicare. Department of Health. 2016. http://www.health.gov.au/internet/main/publishing.nsf/Content/mbsprimarycare_mbsitem_general_factsheet. Accessed 14 Aug 2018.

[14] Humanitarian Settlement Program. Department of Social Services, 2018. https://www.dss.gov.au/settlement-services/programs-policy/settlement-services/humanitarian-settlement-program. Accessed 14 Aug 2018.

[15] Robertshaw L, Dhesi S, Jones LL (2017). Challenges and facilitators for health professionals providing primary healthcare for refugees and asylum seekers in high-income countries: a systematic review and thematic synthesis of qualitative research. BMJ Open.

[16] Humanitarian Settlement in Regional Australia. In: Settlement Services Department of Social Services 2018. https://www.dss.gov.au/settlement-and-multicultural-affairs/publications/humanitarian-settlement-in-regional-australia. Accessed 1 April 2019.

[17] Mangrio E, Sjögren Forss K (2017). Refugees’ experiences of healthcare in the host country: a scoping review. BMC Health Serv Res.

[18] Cheng IH, Drillich A, Schattner P (2015). Refugee experiences of general practice in countries of resettlement: a literature review. Br J Gen Pract.

[19] Valibhoy MC, Szwarc J, Kaplan I (2017). Young service users from refugee backgrounds: their perspectives on barriers to accessing Australian mental health services. Int J Human Rights Healthcare.

[20] Samuel Sophia, Advocat Jenny, Russell Grant (2018). Health seeking narratives of unwell Sri Lankan Tamil refugees in Melbourne Australia. Australian Journal of Primary Health.

[21] Thomas J, Harden A (2008). Methods for the thematic synthesis of qualitative research in systematic reviews. BMC Med Res Methodol.

[22] Liberati A, Altman DG, Tetzlaff J, Mulrow C, Gøtzsche PC, Ioannidis JPA, Clarke M, Devereaux PJ, Kleijnen J, Moher D (2009). The PRISMA statement for reporting systematic reviews and meta-analyses of studies that evaluate health care interventions: explanation and elaboration. PLoS Med.

[23] Tong A, Flemming K, McInnes E, Oliver S, Craig J (2012). Enhancing transparency in reporting the synthesis of qualitative research: ENTREQ. BMC Med Res Methodol.

[24] Toye F, Seers K, Allcock N, Briggs M, Carr E, Barker K (2014). Meta-ethnography 25 years on: challenges and insights for synthesising a large number of qualitative studies. BMC Med Res Methodol.

[25] Butler A, Hall H, Copnell B (2016). A guide to writing a qualitative systematic review protocol to enhance evidence-based practice in nursing and health care. Worldviews Evid-Based Nurs.

[26] Bearman M, Dawson P (2013). Qualitative synthesis and systematic review in health professions education. Med Educ.

[27] Mays N, Pope C (2000). Assessing quality in qualitative research. BMJ..

[28] Bellamy K, Ostini R, Martini N, Kairuz T (2017). Perspectives of resettled African refugees on accessing medicines and pharmacy services in Queensland, Australia. Int J Pharm Pract.

[29] Carolan M, Cassar L (2010). Antenatal care perceptions of pregnant African women attending maternity services in Melbourne, Australia. Midwifery..

[30] Cheng IH, Vasi S, Wahidi S, Russell G (2015). Rites of passage: improving refugee access to general practice services. Aust Fam Physician.

[31] Clark A, Gilbert A, Rao D, Kerr L (2014). 'Excuse me, do any of you ladies speak English?' perspectives of refugee women living in South Australia: barriers to accessing primary health care and achieving the quality use of medicines. Aust J Prim Health.

[32] Correa-Velez I, Ryan J (2012). Developing a best practice model of refugee maternity care. Women Birth.

[33] Henderson S, Kendall E (2011). Culturally and linguistically diverse Peoples' knowledge of accessibility and utilisation of health services: exploring the need for improvement in health service delivery. Aust J Prim Health..

[34] Kay M, Wijayanayaka S, Cook H, Hollingworth S (2016). Understanding quality use of medicines in refugee communities in Australian primary care: a qualitative study. Br J Gen Pract.

[35] Liamputtong P, Watson LF (2006). The meanings and experiences of cesarean birth among Cambodian, Lao and Vietnamese immigrant women in Australia. Women Health.

[36] McBride J, Russo A, Block A (2016). The refugee health nurse liaison: a nurse led initiative to improve healthcare for asylum seekers and refugees. Contemp Nurse.

[37] McBride J, Block A, Russo A (2017). An integrated healthcare service for asylum seekers and refugees in the south-eastern region of Melbourne: Monash health refugee health and wellbeing. Aust J Prim Health..

[38] McCann TV, Mugavin J, Renzaho A, Lubman DI. Sub-Saharan African migrant youths' help-seeking barriers and facilitators for mental health and substance use problems: a qualitative study. BMC Psychiatry. 2016;16:275. 10.1186/s12888-016-0984-5 PMC4971683 27484391

[39] Murray L, Windsor C, Parker E, Tewfik O (2010). The experiences of African women giving birth in Brisbane, Australia. Health Care Women Int.

[40] Neale Andrea, Ngeow Joanne Y. Y., Skull Susan A., Biggs Beverley-Ann (2007). Health services utilisation and barriers for settlers from the Horn of Africa. Australian and New Zealand Journal of Public Health.

[41] Nicol P, Al-Hanbali A, King N, Slack-Smith L, Cherian S. Informing a culturally appropriate approach to oral health and dental care for pre-school refugee children: a community participatory study. BMC Oral Health. 2014;14:69. 10.1186/1472-6831-14-69 PMC4061102 24923308

[42] Niner S, Kokanovic R, Cuthbert D (2013). Displaced mothers: birth and resettlement, gratitude and complaint. Med Anthropol.

[43] O'Callaghan C, Quine S (2007). How older Vietnamese Australian women manage their medicines. J Cross Cult Gerontol.

[44] Omeri A, Lennings C, Raymond L (2006). Beyond asylum: implications for nursing and health care delivery for afghan refugees in Australia. J Transcult Nurs.

[45] Owens C, Dandy J, Hancock P (2016). Perceptions of pregnancy experiences when using a community-based antenatal service: a qualitative study of refugee and migrant women in Perth, Western Australia. Women Birth..

[46] Phillips C (2013). Remote telephone interpretation in medical consultations with refugees: meta-communications about care, survival and selfhood. J Refug Stud.

[47] Riggs E, Davis E, Gibbs L, Block K, Szwarc J, Casey S, Duell-Piening P, Waters E. Accessing maternal and child health services in Melbourne, Australia: reflections from refugee families and service providers. BMC Health Serv Res. 2012;12:117. 10.1186/1472-6963-12-117 PMC3424108 22587587

[48] Riggs E, Yelland J, Szwarc J, Wahidi S, Casey S, Chesters D, Fouladi F, Duell-Piening P, Giallo R, Brown S (2016). Fatherhood in a new country: a qualitative study exploring the experiences of afghan men and implications for health services. Birth..

[49] Riggs E, Muyeen S, Brown S, Dawson W, Petschel P, Tardiff W, Norman F, Vanpraag D, Szwarc J, Yelland J (2017). Cultural safety and belonging for refugee background women attending group pregnancy care: an Australian qualitative study. Birth..

[50] Russo A, Lewis B, Joyce A, Crockett B, Luchters S. A qualitative exploration of the emotional wellbeing and support needs of new mothers from Afghanistan living in Melbourne, Australia. BMC Pregnancy Childbirth. 2015;15:197. 10.1186/s12884-015-0631-z PMC4552986 26319482

[51] Sheikh M, Nugus PI, Gao Z, Holdgate A, Short AE, Al Haboub A, Raina MacIntyre C (2011). Equity and access: understanding emergency health service use by newly arrived refugees. Med J Aust.

[52] Sievert K, O’Neill P, Koh Y, Lee JH, Dev A, Le S (2018). Barriers to accessing testing and treatment for chronic hepatitis B in afghan, Rohingyan, and south Sudanese populations in Australia. J Immigr Minor Health.

[53] Stapleton H, Murphy R, Correa-Velez I, Steel M, Kildea S (2013). Women from refugee backgrounds and their experiences of attending a specialist antenatal clinic. Narratives from an Australian setting. Women Birth..

[54] Sypek S, Clugston G, Phillips C (2008). Critical health infrastructure for refugee resettlement in rural Australia: case study of four rural towns. Aust J Rural Health.

[55] Valibhoy MC, Kaplan I, Szwarc J (2017). "it comes down to just how human someone can be": a qualitative study with young people from refugee backgrounds about their experiences of Australian mental health services. Transcult Psychiatry.

[56] Wohler Y, Dantas JA (2017). Barriers accessing mental health services among culturally and linguistically diverse (CALD) immigrant women in Australia: policy implications. J Immigr Minor Health.

[57] Yelland J, Riggs E, Wahidi S, Fouladi F, Casey S, Szwarc J, Duell-Piening P, Chesters D, Brown S. How do Australian maternity and early childhood health services identify and respond to the settlement experience and social context of refugee background families? BMC Pregnancy Childbirth. 2014;14:348. 10.1186/1471-2393-14-348 PMC4287513 25284336

[58] Yelland J, Riggs E, Szwarc J, Casey S, Duell-Piening P, Chesters D, Wahidi S, Fouladi F, Brown S (2016). Compromised communication: a qualitative study exploring afghan families and health professionals' experience of interpreting support in Australian maternity care. BMJ Qual Saf.

[59] Riggs E, Yelland J, Shankumar R, Kilpatrick N. 'We are all scared for the baby': promoting access to dental services for refugee background women during pregnancy. BMC Pregnancy Childbirth. 2016;16(1):12. 10.1186/s12884-015-0787-6 PMC4722780 26794243

[60] Robards F, Kang M, Steinbeck K, Hawke C, Jan S, Sanci L, Liew YY, Kong M, Usherwood T (2019). Health care equity and access for marginalised young people: a longitudinal qualitative study exploring health system navigation in Australia. Int J Equity Health.

[61] A New Definition of Patient Engagement: What is Engagement and Why is it Important? Washington DC, United States of America: Centre for Advancing Health, Washington DC, United States of America. 2010. http://www.cfah.org/file/CFAH_Engagement_Behavior_Framework_current.pdf. Accessed 28 Aug 2018.

[62] C Ratzan S, Parker R, R Selden C, Zorn M. National library of medicine current bibliographies in medicine: health Literacy; 2000.

[63] Penchansky R, Thomas JW (1981). The concept of access: definition and relationship to consumer satisfaction. Med Care.

[64] Dixon-Woods M, Shaw R, Agarwal S, Smith J (2004). The problem of appraising qualitative research. Qual Saf Health Care.

[65] Queensland Language Services Policy. Queensland, Australia: Department of Communities, child safety and disability services, Queensland, Australia. 2016. http://www.dlgrma.qld.gov.au/resources/multicultural/policy-governance/lsp-policy.pdf. Accessed 20 Aug 2018.

[66] Interpreters – Standard Procedures for Working with Health Care Interpreters. New South Wales, Australia: NSW health, New South Wales, Australia. 2017. https://www1.health.nsw.gov.au/pds/ActivePDSDocuments/PD2017_044.pdf. Accessed 20 Aug 2018.

[67] Language services policy. State of Victoria, Department of health and human services, 2017. https://dhhs.vic.gov.au/sites/default/files/documents/201703/DHHS-Language-services-policy-January-2017_0.docx. Accessed 20 Aug 2018.

[68] Phillips CB, Travaglia J (2011). Low levels of uptake of free interpreters by Australian doctors in private practice: secondary analysis of national data. Aust Health Rev.

[69] Truong M, Paradies Y, Priest N (2014). Interventions to improve cultural competency in healthcare: a systematic review of reviews. BMC Health Serv Res.

[70] Griswold KS (2003). Refugee health and medical student training. Fam Med.

[71] Dussán KB, Galbraith EM, Grzybowski M, Vautaw BM, Murray L, Eagle KA (2009). Effects of a refugee elective on medical student perceptions. BMC Med Educ.

[72] Ogunsiji Olayide, Ng Chok Harrison, Mashingaidze Gladys, Wilkes Lesley (2017). “I am still passionate despite the challenges”: Nurses navigating the care for refugees. Journal of Clinical Nursing.

[73] Farley R, Askew D, Kay M (2014). Caring for refugees in general practice: perspectives from the coalface. Aust J Prim Health..

[74] Milosevic D, Cheng IH, Smith MM (2012). The NSW refugee health service: improving refugee access to primary care. Aust Fam Physician.

[75] Farr M, Cressey P (2015). Understanding staff perspectives of quality in practice in healthcare. BMC Health Serv Res.

[76] Leung L (2015). Validity, reliability, and generalizability in qualitative research. J Family Med Prim Care.

[77] Booth A, Lewin S, Glenton C, Munthe-Kaas H, Toews I, Noyes J, Rashidian A, Berg RC, Nyakang’o B, Meerpohl JJ (2018). Applying GRADE-CERQual to qualitative evidence synthesis findings–paper 7: understanding the potential impacts of dissemination bias. Implement Sci.

