# Supplementary material for: A model explaining refugee experiences of the Australian healthcare system: a systematic review of refugee perceptions
Source: BMC Int Health Hum Rights. 2019 Jul 18;19:22. doi: 10.1186/s12914-019-0206-6 (PMC6637597; doi:10.1186/s12914-019-0206-6)
Supplement: Supplementary file 1 — Search Strategy. Search strategy used in Scopus and MEDLINE. (DOCX 23 kb) [file 12914_2019_206_MOESM1_ESM.docx]

**Appendix 1**

Search Strategy

The following is our search strategy for Scopus:

refugee* OR "asylum seeker*" OR migrant* OR immigrant*

AND

"health service*" OR hospital* OR "community health" OR "health system*" OR "health outcome*"

AND

austral* OR nsw OR "New South Wales" OR qld OR queensland* OR vic OR victoria* OR sa OR "South Australia*" OR wa OR "Western Australia*" OR tas OR tasmania* OR nt OR "Northern Territory" OR act OR “Australian capital territory”

AND

“health outcome*” OR satisf* OR perce* OR experience* OR expect* OR challenge* OR barrier* OR engagement OR “cultural competence” OR communication* OR enabler* OR access* OR issue* OR facilita* OR preference* OR narrative* OR promoter*

Limits: Geography to Australia and New Zealand

Our search strategy on MEDLINE was the following:

| 1 | exp Refugees/ |
| --- | --- |
| 2 | exp "Transients and Migrants"/ or "asylum seeker*".mp. |
| 3 | exp "Emigration and Immigration"/ or migrant*.mp. or exp "Emigrants and Immigrants"/ |
| 4 | exp UNDOCUMENTED IMMIGRANTS/ or exp "EMIGRANTS AND IMMIGRANTS"/ or immigrant*.mp. |
| 5 | 1 or 2 or 3 or 4 |
| 6 | exp Mental Health Services/ or exp "Delivery of Health Care"/ or "health service*".mp. |
| 7 | hospital*.mp. or exp HOSPITAL DEPARTMENTS/ or exp EMERGENCY SERVICE, HOSPITAL/ |
| 8 | exp Primary Health Care/ or exp Mental Health Services/ or exp "Delivery of Health Care"/ or "health system*".mp. |
| 9 | exp Health Status/ or "health outcome*".mp. |
| 10 | "community health".mp. or Public Health/ |
| 11 | 6 or 7 or 8 or 9 or 10 |
| 12 | exp SOUTH AUSTRALIA/ or Australia.mp. or exp AUSTRALIA/ or exp WESTERN AUSTRALIA/ |
| 13 | satisf*.mp. |
| 14 | exp Perception/ or perce*.mp. |
| 15 | experience*.mp. |
| 16 | expect*.mp. |
| 17 | challenge*.mp. |
| 18 | barrier*.mp. |
| 19 | engagement.mp. |
| 20 | cultural competence.mp. or exp Cultural Competency/ |
| 21 | communication*.mp. or COMMUNICATION/ or HEALTH COMMUNICATION/ |
| 22 | enabler*.mp. |
| 23 | exp ACCESS TO INFORMATION/ or access*.mp. |
| 24 | issue*.mp. |
| 25 | facilita*.mp. |
| 26 | exp PATIENT PREFERENCE/ or preference*.mp. |
| 27 | narrative*.mp. |
| 28 | promoter*.mp. |
| 29 | 9 or 13 or 14 or 15 or 16 or 17 or 18 or 19 or 20 or 21 or 22 or 23 or 24 or 25 or 26 or 27 or 28 |
| 30 | 5 and 11 and 12 and 29 |

Limits: Geography to Australia and New Zealand
