# Supplementary material for: A model explaining refugee experiences of the Australian healthcare system: a systematic review of refugee perceptions
Source: BMC Int Health Hum Rights. 2019 Jul 18;19:22. doi: 10.1186/s12914-019-0206-6 (PMC6637597; doi:10.1186/s12914-019-0206-6)
Supplement: Supplementary file 2 — Modified Critical Appraisal Tool (MCAT). A standardised qualitative appraisal tool used by authors to appraise studies with qualitative data. (DOCX 26 kb) [file 12914_2019_206_MOESM2_ESM.docx]

**Appendix 2**

Modified Critical Appraisal Tool (MCAT)

*Instruments developed to support quality appraisal usually share some basic criteria for the assessment of qualitative research. These include the need for research to have been conducted ethically, the consideration of relevance to inform practice or policy, the use of appropriate and rigorous methods and the clarity and coherence of reporting (Cohen & Crabtree, 2008).*

| THEORETICAL APPRAISAL | CONSIDERATIONS | REFERENCES |
| --- | --- | --- |
| Is there congruity between the stated philosophical perspective and the research methodology?  Example of method:   - Phenomenology - Ethnography - Grounded theory - Participatory action research   Example of philosophical perspective:   - Positivism - Realism - Interpretivism | With the decision of the philosophical perspective, consider the quality of the decisions made, the rationale behind them or the responsiveness or sensibility of the researcher to the data. Also consider an evaluation of methodological coherence or congruity between paradigms that guide the research project and the methodology and methods chosen, an active analytic stance and theoretical position, investigator responsiveness and openness and verification, which refers to systematically checking and confirming the fit between data gathered and the conceptual work of analysis and interpretation (Morse et al, 2002). | Hannes K. Chapter 4: Critical appraisal of qualitative research.  JBI QARI  McMaster  Deborah J. Cohen, PhD and Benjamin F. Crabtree, PhD |
| FUNDAMENTALS | CONSIDERATIONS | REFERENCES |
| Is it ethical? |  | CASP  JBI QARI  Deborah J. Cohen, PhD and Benjamin F. Crabtree, PhD |
| Is it important? | - Was there background research done (literature review)? - Describe the justification of the need for this study. Was it clear and compelling? - How valuable is the research? - Research is considered important when it was pragmatically and theoretically useful and advanced the current knowledge base | CASP  Deborah J. Cohen, PhD and Benjamin F. Crabtree, PhD  McMaster |
| REPORTING | CONSIDERATIONS | REFERENCES |
| Does it meet most of the SRQR (Standards for reporting qualitative research) Checklist? What are the omissions? | - Refer to Standards for Reporting Qualitative Research (SRQR) <http://www.equator-network.org/reporting-guidelines/srqr/> | SRQR |
| Is there clarity and coherence of the research report? | Clarity and coherence of the research report were criteria emphasizing that the report itself should be concise and provide a clear and adequate description of the research question, background and contextual material, study design (e.g., study participants, how they were chosen, how data are collected and analysed), and rationale for methodological choices. Description of the data should be unexaggerated, and the relationship between data and interpretation should be understandable. | JBI QARI  Deborah J. Cohen, PhD and Benjamin F. Crabtree, PhD |
| CREDIBILITY (Validity) | CONSIDERATIONS | REFERENCES |
| Were the research methods appropriate to reach the study’s goals? |  | CASP  McMaster  JBI QARI |
| Was the recruitment strategy appropriate for the aims of the research? | - If they explained why the participants they selected were the most appropriate to provide access to the type of knowledge sought by the study? - If there are any discussions around recruitment (e.g. why some people chose not to take part) | CASP  McMaster |
| Was the data collected in a way that addressed the research issue? | - If the setting for data collection was justified - If it is clear how data were collected (e.g. focus group, semi-structured interview etc.) - If the researcher has justified the methods chosen - If the form of data is clear (e.g. tape recordings, video material, notes etc) - If the researcher has discussed saturation of data | CASP  JBI QARI  McMaster |
| Is researcher bias addressed in the study design, site chosen and recruitment? | - Does the article provide background information on the researcher’s background, education, perspective, school of thought etc? - How did the researcher respond to this? - Is there a statement locating the researcher culturally or theoretically? - Is the influence of the researcher on the research and vice-versa addressed? | CASP  JBI QARI |
| How was the data interpreted? |  | JBI QARI |
| Was the data analysis rigorous?  (Confirmability) | - If there is an in-depth description of the analysis process - If thematic analysis is used. If so, is it clear how the categories/themes were derived from the data? - Whether the researcher explains how the data presented were selected from the original sample to demonstrate the analysis process - If sufficient data are presented to support the findings - To what extent contradictory data are taken into account - Whether the researcher critically examined their own role, potential bias and influence during analysis and selection of data for presentation - Are multiple researchers involved? | CASP  JBI QARI  McMaster |
| Is the data adequately represented? | - Are participants and their voices adequately represented? | JBI QARI |
| DEPENDABILITY | CONSIDERATIONS | REFERENCES |
| - Triangulation - Peer review/ debriefing - External audits/ auditing - Member checking/respondent validation - Reflexivity - Multiple coding - Deviant case analysis   *With respect to appropriateness* | Some methods may not be appropriate for all studies. | Hannes K. Chapter 4: Critical appraisal of qualitative research.  Deborah J. Cohen, PhD and Benjamin F. Crabtree, PhD |
| Is researcher bias addressed in the data collection? | - Does the article provide background information on the researcher’s background, education, perspective, school of thought etc? - How did the researcher respond to this? - Is there a statement locating the researcher culturally or theoretically? - Is the influence of the researcher on the research and vice-versa addressed? | CASP  JBI QARI |
| UTILITY | CONSIDERATIONS | REFERENCES |
| Is the study relevant to our research question? | - Consider our inclusion/exclusion criteria. - Are conclusions relevant to our research? | Hannes K. Chapter 4: Critical appraisal of qualitative research. |
| Is the phenomena of interest represented adequately by the researcher’s findings? | - Consider generalisability of results. | Hannes K. Chapter 4: Critical appraisal of qualitative research. |
